# 

**Published:** 1888-05

**Authors:** 


					﻿Volume LVI. |
MAY, 1888.
' Number 5«
The CHICAGO
TMEDK-AL:
? Journal
EXAMINER
EDITOR:
S. J. JONES, M.D., LED
Rand, McNally & Company, Printers.
1888,
Entered at the Post Office at Chicago, for transmisaion through the mails as (•■•nd-olass matter.
				

